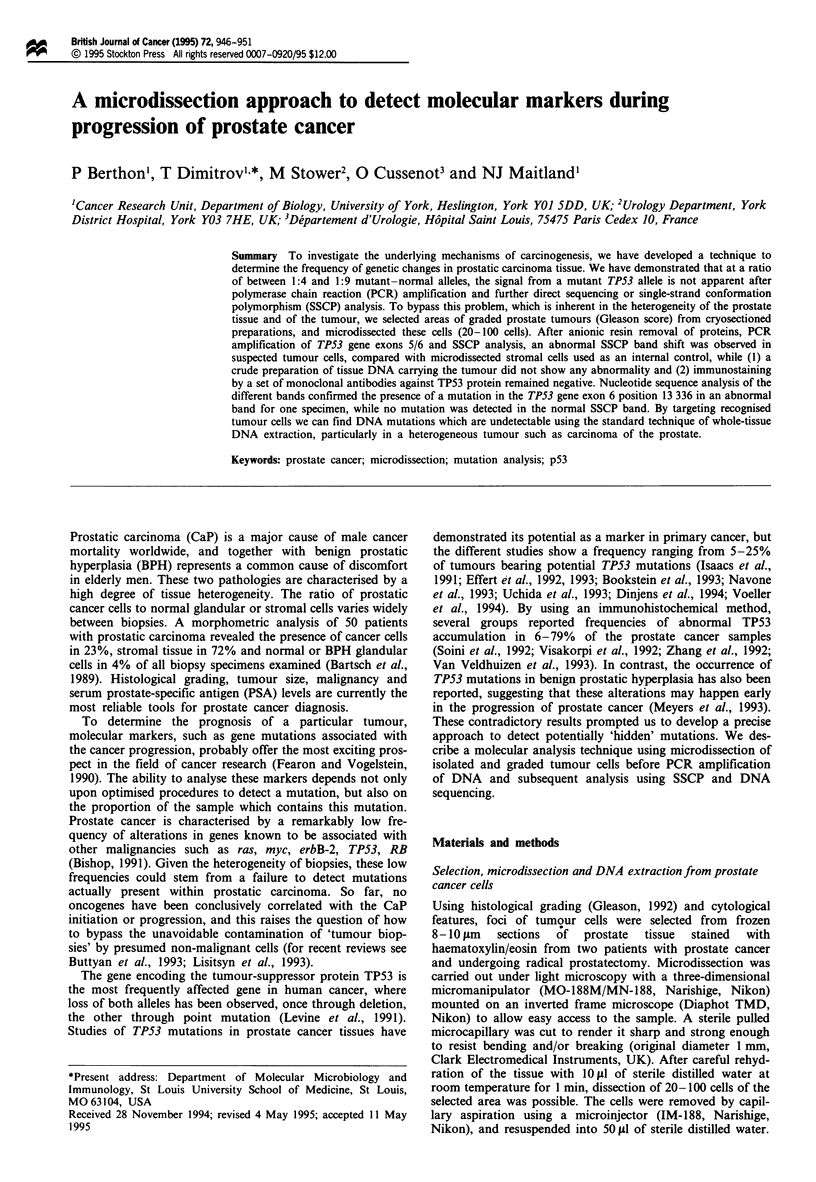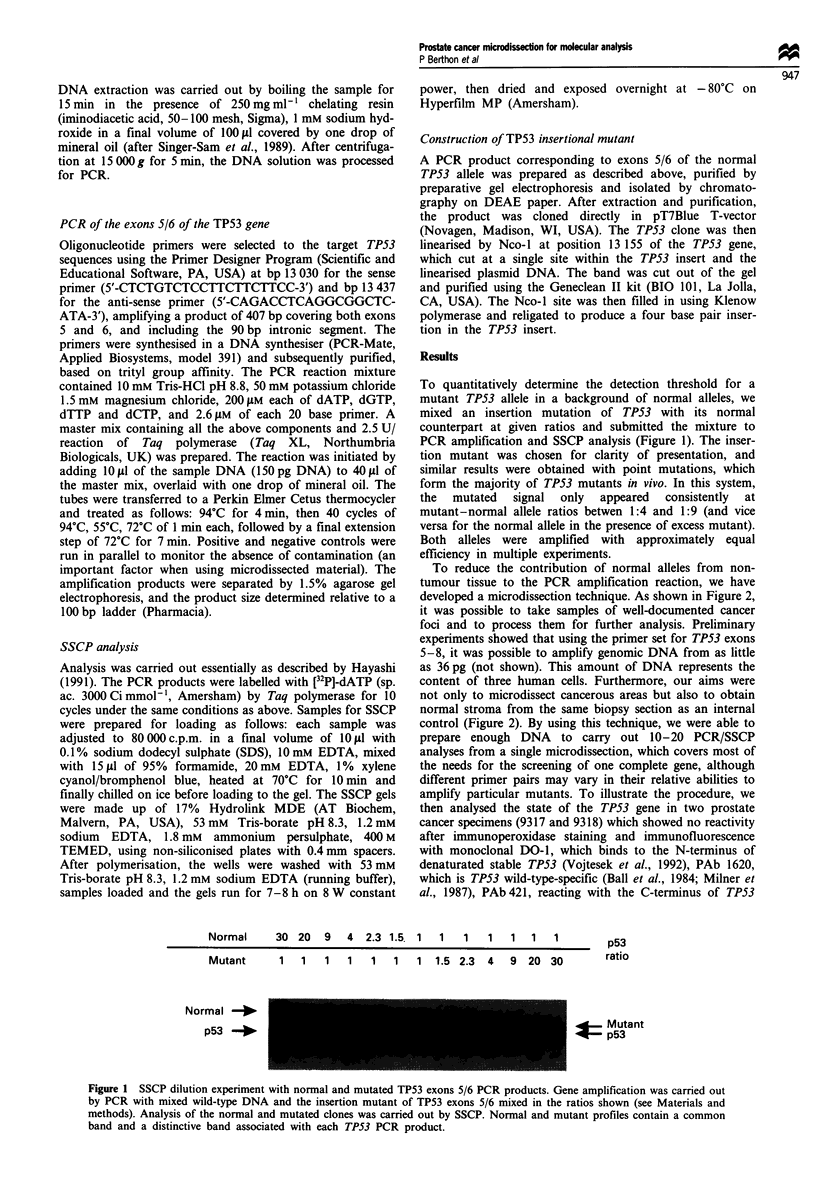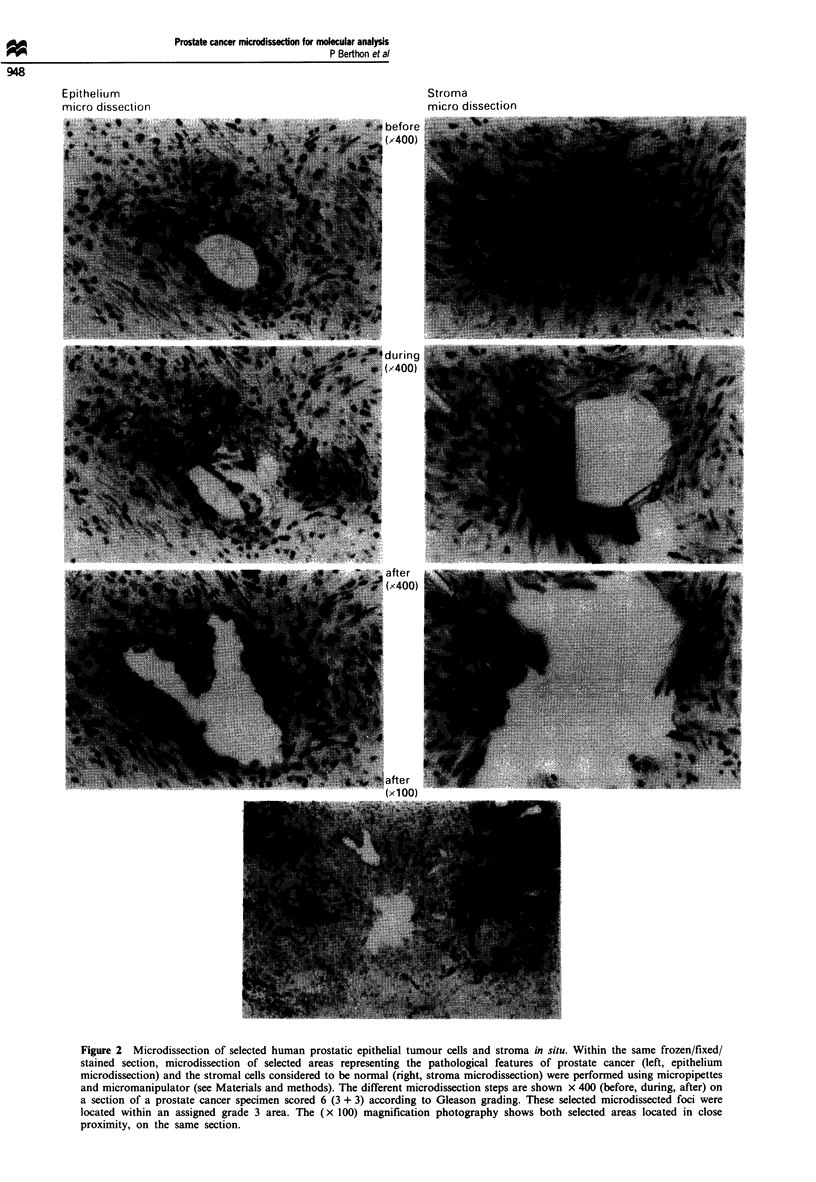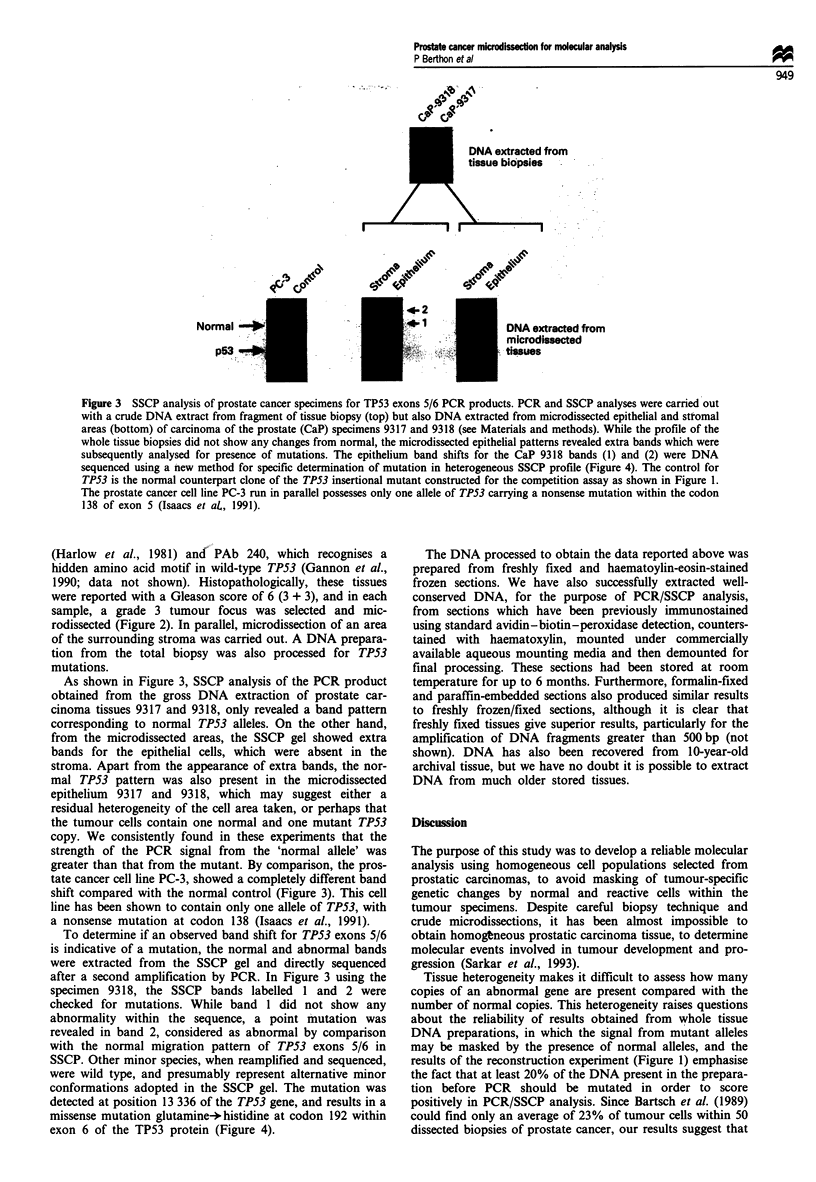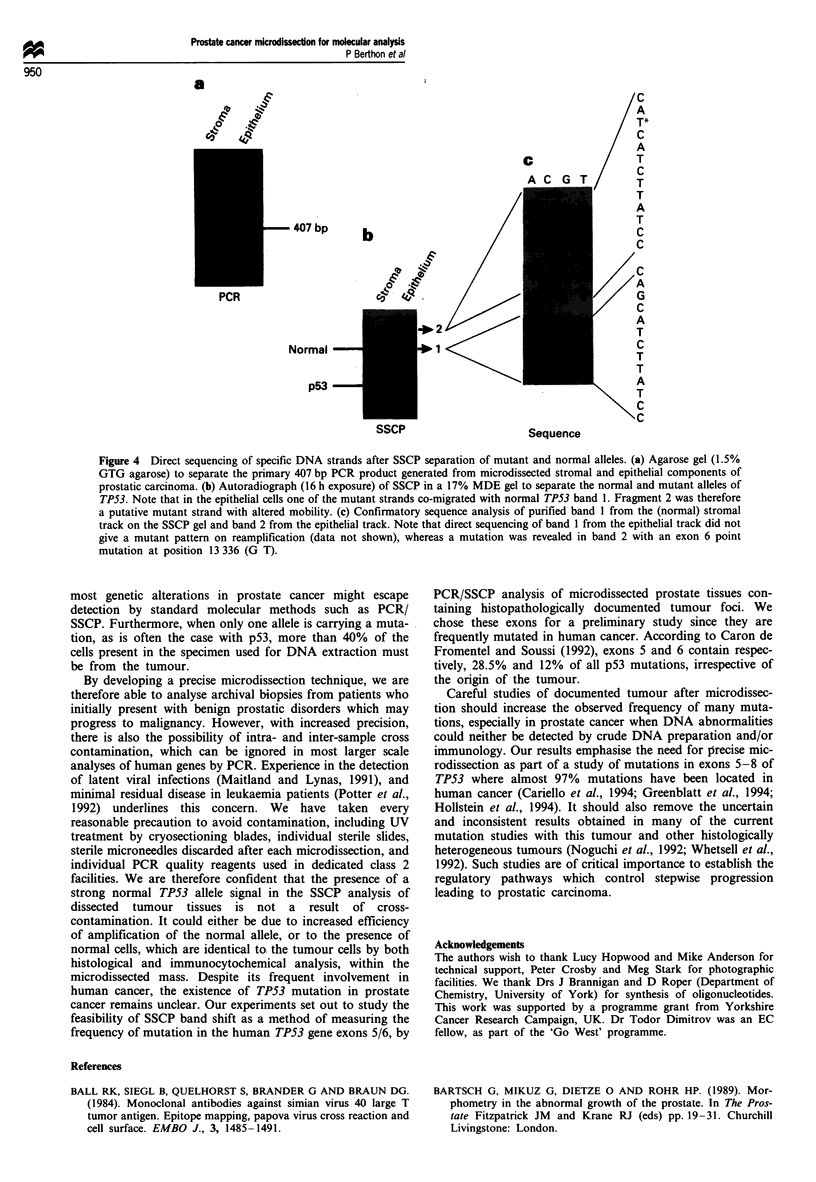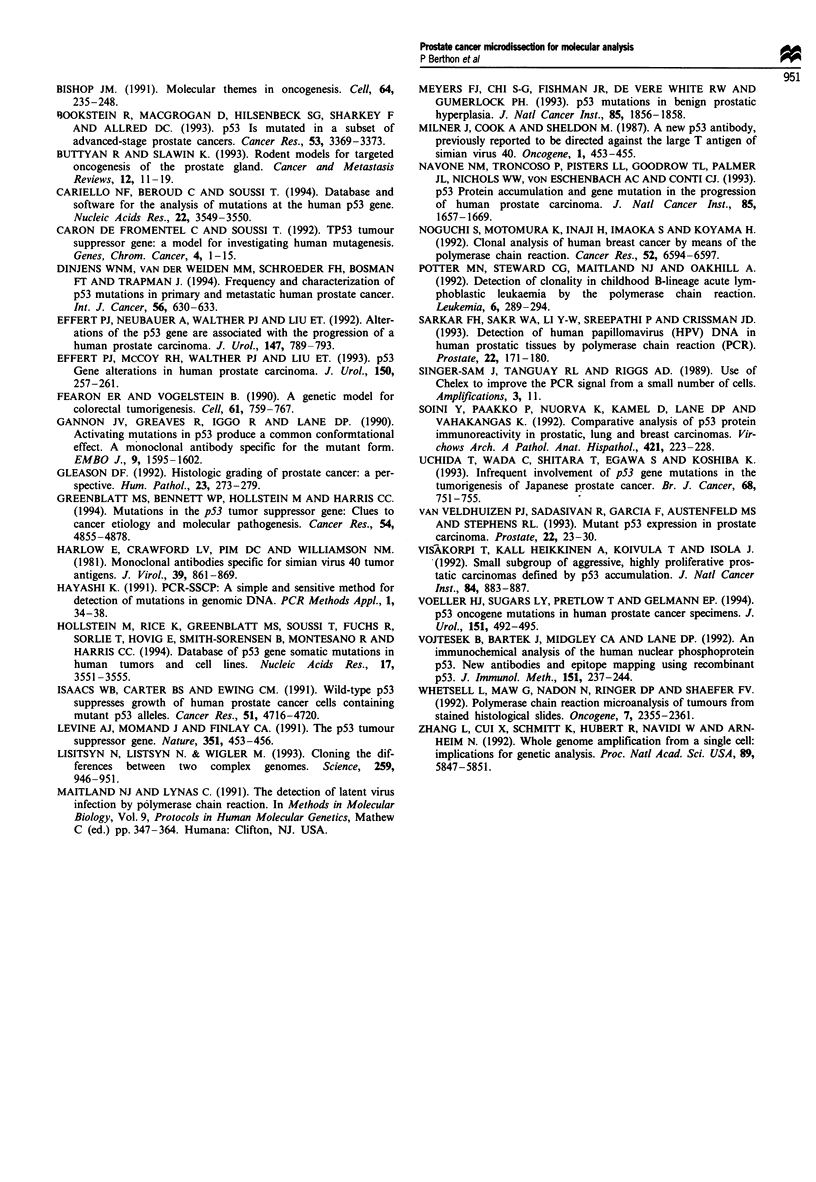# A microdissection approach to detect molecular markers during progression of prostate cancer.

**DOI:** 10.1038/bjc.1995.439

**Published:** 1995-10

**Authors:** P. Berthon, T. Dimitrov, M. Stower, O. Cussenot, N. J. Maitland

**Affiliations:** Department of Biology, University of York, Heslington, UK.

## Abstract

**Images:**


British Journal of Cancer (1995) 72, 946-951
© 1995 Stockton Press All rights reserved 0007-0920/95 $12.00

### A microdissection approach to detect molecular markers during progression of prostate cancer

P Berthon[1], T Dimitrov[1,*], M Stower[2], O Cussenot[3] and NJ Maitland[1]

[1]*Cancer Research Unit, Department of Biology, University of York, Heslington, York Y01 5DD, UK;* [2]*Urology Department, York District Hospital, York Y03 7HE, UK;* [3]*Département d'Urologie, Hôpital Saint Louis, 75475 Paris Cedex 10, France*

**Summary** To investigate the underlying mechanisms of carcinogenesis, we have developed a technique to determine the frequency of genetic changes in prostatic carcinoma tissue. We have demonstrated that at a ratio of between 1:4 and 1:9 mutant–normal alleles, the signal from a mutant *TP53* allele is not apparent after polymerase chain reaction (PCR) amplification and further direct sequencing or single-strand conformation polymorphism (SSCP) analysis. To bypass this problem, which is inherent in the heterogeneity of the prostate tissue and of the tumour, we selected areas of graded prostate tumours (Gleason score) from cryosectioned preparations, and microdissected these cells (20–100 cells). After anionic resin removal of proteins, PCR amplification of *TP53* gene exons 5/6 and SSCP analysis, an abnormal SSCP band shift was observed in suspected tumour cells, compared with microdissected stromal cells used as an internal control, while (1) a crude preparation of tissue DNA carrying the tumour did not show any abnormality and (2) immunostaining by a set of monoclonal antibodies against TP53 protein remained negative. Nucleotide sequence analysis of the different bands confirmed the presence of a mutation in the *TP53* gene exon 6 position 13 336 in an abnormal band for one specimen, while no mutation was detected in the normal SSCP band. By targeting recognised tumour cells we can find DNA mutations which are undetectable using the standard technique of whole-tissue DNA extraction, particularly in a heterogeneous tumour such as carcinoma of the prostate.

**Keywords:** prostate cancer; microdissection; mutation analysis; p53

Prostatic carcinoma (CaP) is a major cause of male cancer mortality worldwide, and together with benign prostatic hyperplasia (BPH) represents a common cause of discomfort in elderly men. These two pathologies are characterised by a high degree of tissue heterogeneity. The ratio of prostatic cancer cells to normal glandular or stromal cells varies widely between biopsies. A morphometric analysis of 50 patients with prostatic carcinoma revealed the presence of cancer cells in 23%, stromal tissue in 72% and normal or BPH glandular cells in 4% of all biopsy specimens examined (Bartsch *et al.*, 1989). Histological grading, tumour size, malignancy and serum prostate-specific antigen (PSA) levels are currently the most reliable tools for prostate cancer diagnosis.

To determine the prognosis of a particular tumour, molecular markers, such as gene mutations associated with the cancer progression, probably offer the most exciting prospect in the field of cancer research (Fearon and Vogelstein, 1990). The ability to analyse these markers depends not only upon optimised procedures to detect a mutation, but also on the proportion of the sample which contains this mutation. Prostate cancer is characterised by a remarkably low frequency of alterations in genes known to be associated with other malignancies such as *ras*, *myc*, *erb*B-2, *TP53*, *RB* (Bishop, 1991). Given the heterogeneity of biopsies, these low frequencies could stem from a failure to detect mutations actually present within prostatic carcinoma. So far, no oncogenes have been conclusively correlated with the CaP initiation or progression, and this raises the question of how to bypass the unavoidable contamination of 'tumour biopsies' by presumed non-malignant cells (for recent reviews see Buttyan *et al.*, 1993; Lisitsyn *et al.*, 1993).

The gene encoding the tumour-suppressor protein TP53 is the most frequently affected gene in human cancer, where loss of both alleles has been observed, once through deletion, the other through point mutation (Levine *et al.*, 1991). Studies of *TP53* mutations in prostate cancer tissues have demonstrated its potential as a marker in primary cancer, but the different studies show a frequency ranging from 5–25% of tumours bearing potential *TP53* mutations (Isaacs *et al.*, 1991; Effert *et al.*, 1992, 1993; Bookstein *et al.*, 1993; Navone *et al.*, 1993; Uchida *et al.*, 1993; Dinjens *et al.*, 1994; Voeller *et al.*, 1994). By using an immunohistochemical method, several groups reported frequencies of abnormal TP53 accumulation in 6–79% of the prostate cancer samples (Soini *et al.*, 1992; Visakorpi *et al.*, 1992; Zhang *et al.*, 1992; Van Veldhuizen *et al.*, 1993). In contrast, the occurrence of *TP53* mutations in benign prostatic hyperplasia has also been reported, suggesting that these alterations may happen early in the progression of prostate cancer (Meyers *et al.*, 1993). These contradictory results prompted us to develop a precise approach to detect potentially 'hidden' mutations. We describe a molecular analysis technique using microdissection of isolated and graded tumour cells before PCR amplification of DNA and subsequent analysis using SSCP and DNA sequencing.

### Materials and methods

### *Selection, microdissection and DNA extraction from prostate cancer cells*

Using histological grading (Gleason, 1992) and cytological features, foci of tumour cells were selected from frozen 8–10 μm sections of prostate tissue stained with haematoxylin/eosin from two patients with prostate cancer and undergoing radical prostatectomy. Microdissection was carried out under light microscopy with a three-dimensional micromanipulator (MO-188M/MN-188, Narishige, Nikon) mounted on an inverted frame microscope (Diaphot TMD, Nikon) to allow easy access to the sample. A sterile pulled microcapillary was cut to render it sharp and strong enough to resist bending and/or breaking (original diameter 1 mm, Clark Electromedical Instruments, UK). After careful rehydration of the tissue with 10 μl of sterile distilled water at room temperature for 1 min, dissection of 20–100 cells of the selected area was possible. The cells were removed by capillary aspiration using a microinjector (IM-188, Narishige, Nikon), and resuspended into 50 μl of sterile distilled water.

*Present address: Department of Molecular Microbiology and Immunology, St Louis University School of Medicine, St Louis, MO 63104, USA
Received 28 November 1994; revised 4 May 1995; accepted 11 May 1995

DNA extraction was carried out by boiling the sample for 15 min in the presence of 250 mg ml$^{-1}$ chelating resin (iminodiacetic acid, 50–100 mesh, Sigma), 1 mM sodium hydroxide in a final volume of 100 μl covered by one drop of mineral oil (after Singer-Sam *et al*., 1989). After centrifugation at 15 000 *g* for 5 min, the DNA solution was processed for PCR.

### *PCR of the exons 5/6 of the* TP53 *gene*

Oligonucleotide primers were selected to the target *TP53* sequences using the Primer Designer Program (Scientific and Educational Software, PA, USA) at bp 13 030 for the sense primer (5′-CTCTGTCTCCTTCTTCTTCC-3′) and bp 13 437 for the anti-sense primer (5′-CAGACCTCAGGCGGCTC-ATA-3′), amplifying a product of 407 bp covering both exons 5 and 6, and including the 90 bp intronic segment. The primers were synthesised in a DNA synthesiser (PCR-Mate, Applied Biosystems, model 391) and subsequently purified, based on trityl group affinity. The PCR reaction mixture contained 10 mM Tris-HCl pH 8.8, 50 mM potassium chloride 1.5 mM magnesium chloride, 200 μM each of dATP, dGTP, dTTP and dCTP, and 2.6 μM of each 20 base primer. A master mix containing all the above components and 2.5 U/reaction of *Taq* polymerase (*Taq* XL, Northumbria Biologicals, UK) was prepared. The reaction was initiated by adding 10 μl of the sample DNA (150 pg DNA) to 40 μl of the master mix, overlaid with one drop of mineral oil. The tubes were transferred to a Perkin Elmer Cetus thermocycler and treated as follows: 94°C for 4 min, then 40 cycles of 94°C, 55°C, 72°C of 1 min each, followed by a final extension step of 72°C for 7 min. Positive and negative controls were run in parallel to monitor the absence of contamination (an important factor when using microdissected material). The amplification products were separated by 1.5% agarose gel electrophoresis, and the product size determined relative to a 100 bp ladder (Pharmacia).

### *SSCP analysis*

Analysis was carried out essentially as described by Hayashi (1991). The PCR products were labelled with [$^{32}$P]-dATP (sp. ac. 3000 Ci mmol$^{-1}$, Amersham) by *Taq* polymerase for 10 cycles under the same conditions as above. Samples for SSCP were prepared for loading as follows: each sample was adjusted to 80 000 c.p.m. in a final volume of 10 μl with 0.1% sodium dodecyl sulphate (SDS), 10 mM EDTA, mixed with 15 μl of 95% formamide, 20 mM EDTA, 1% xylene cyanol/bromphenol blue, heated at 70°C for 10 min and finally chilled on ice before loading to the gel. The SSCP gels were made up of 17% Hydrolink MDE (AT Biochem, Malvern, PA, USA), 53 mM Tris-borate pH 8.3, 1.2 mM sodium EDTA, 1.8 mM ammonium persulphate, 400 M TEMED, using non-siliconised plates with 0.4 mm spacers. After polymerisation, the wells were washed with 53 mM Tris-borate pH 8.3, 1.2 mM sodium EDTA (running buffer), samples loaded and the gels run for 7–8 h on 8 W constant power, then dried and exposed overnight at −80°C on Hyperfilm MP (Amersham).

### *Construction of* TP53 *insertional mutant*

A PCR product corresponding to exons 5/6 of the normal *TP53* allele was prepared as described above, purified by preparative gel electrophoresis and isolated by chromatography on DEAE paper. After extraction and purification, the product was cloned directly in pT7Blue T-vector (Novagen, Madison, WI, USA). The *TP53* clone was then linearised by Nco-1 at position 13 155 of the *TP53* gene, which cut at a single site within the *TP53* insert and the linearised plasmid DNA. The band was cut out of the gel and purified using the Geneclean II kit (BIO 101, La Jolla, CA, USA). The Nco-1 site was then filled in using Klenow polymerase and religated to produce a four base pair insertion in the *TP53* insert.

### Results

To quantitatively determine the detection threshold for a mutant *TP53* allele in a background of normal alleles, we mixed an insertion mutation of *TP53* with its normal counterpart at given ratios and submitted the mixture to PCR amplification and SSCP analysis (Figure 1). The insertion mutant was chosen for clarity of presentation, and similar results were obtained with point mutations, which form the majority of *TP53* mutants *in vivo*. In this system, the mutated signal only appeared consistently at mutant–normal allele ratios between 1:4 and 1:9 (and vice versa for the normal allele in the presence of excess mutant). Both alleles were amplified with approximately equal efficiency in multiple experiments.

To reduce the contribution of normal alleles from non-tumour tissue to the PCR amplification reaction, we have developed a microdissection technique. As shown in Figure 2, it was possible to take samples of well-documented cancer foci and to process them for further analysis. Preliminary experiments showed that using the primer set for *TP53* exons 5–8, it was possible to amplify genomic DNA from as little as 36 pg (not shown). This amount of DNA represents the content of three human cells. Furthermore, our aims were not only to microdissect cancerous areas but also to obtain normal stroma from the same biopsy section as an internal control (Figure 2). By using this technique, we were able to prepare enough DNA to carry out 10–20 PCR/SSCP analyses from a single microdissection, which covers most of the needs for the screening of one complete gene, although different primer pairs may vary in their relative abilities to amplify particular mutants. To illustrate the procedure, we then analysed the state of the *TP53* gene in two prostate cancer specimens (9317 and 9318) which showed no reactivity after immunoperoxidase staining and immunofluorescence with monoclonal DO-1, which binds to the N-terminus of denaturated stable *TP53* (Vojtesek *et al*., 1992), PAb 1620, which is *TP53* wild-type-specific (Ball *et al*., 1984; Milner *et al*., 1987), PAb 421, reacting with the C-terminus of *TP53*

**Figure 1** SSCP dilution experiment with normal and mutated TP53 exons 5/6 PCR products. Gene amplification was carried out by PCR with mixed wild-type DNA and the insertion mutant of TP53 exons 5/6 mixed in the ratios shown (see Materials and methods). Analysis of the normal and mutated clones was carried out by SSCP. Normal and mutant profiles contain a common band and a distinctive band associated with each *TP53* PCR product.

**Figure 2** Microdissection of selected human prostatic epithelial tumour cells and stroma *in situ*. Within the same frozen/fixed/stained section, microdissection of selected areas representing the pathological features of prostate cancer (left, epithelium microdissection) and the stromal cells considered to be normal (right, stroma microdissection) were performed using micropipettes and micromanipulator (see Materials and methods). The different microdissection steps are shown × 400 (before, during, after) on a section of a prostate cancer specimen scored 6 (3 + 3) according to Gleason grading. These selected microdissected foci were located within an assigned grade 3 area. The (× 100) magnification photography shows both selected areas located in close proximity, on the same section.

**Figure 3** SSCP analysis of prostate cancer specimens for TP53 exons 5/6 PCR products. PCR and SSCP analyses were carried out with a crude DNA extract from fragment of tissue biopsy (top) but also DNA extracted from microdissected epithelial and stromal areas (bottom) of carcinoma of the prostate (CaP) specimens 9317 and 9318 (see Materials and methods). While the profile of the whole tissue biopsies did not show any changes from normal, the microdissected epithelial patterns revealed extra bands which were subsequently analysed for presence of mutations. The epithelium band shifts for the CaP 9318 bands (1) and (2) were DNA sequenced using a new method for specific determination of mutation in heterogeneous SSCP profile (Figure 4). The control for *TP53* is the normal counterpart clone of the *TP53* insertional mutant constructed for the competition assay as shown in Figure 1. The prostate cancer cell line PC-3 run in parallel possesses only one allele of *TP53* carrying a nonsense mutation within the codon 138 of exon 5 (Isaacs *et al.*, 1991).

(Harlow *et al.*, 1981) and PAb 240, which recognises a hidden amino acid motif in wild-type *TP53* (Gannon *et al.*, 1990; data not shown). Histopathologically, these tissues were reported with a Gleason score of 6 (3 + 3), and in each sample, a grade 3 tumour focus was selected and microdissected (Figure 2). In parallel, microdissection of an area of the surrounding stroma was carried out. A DNA preparation from the total biopsy was also processed for *TP53* mutations.

As shown in Figure 3, SSCP analysis of the PCR product obtained from the gross DNA extraction of prostate carcinoma tissues 9317 and 9318, only revealed a band pattern corresponding to normal *TP53* alleles. On the other hand, from the microdissected areas, the SSCP gel showed extra bands for the epithelial cells, which were absent in the stroma. Apart from the appearance of extra bands, the normal *TP53* pattern was also present in the microdissected epithelium 9317 and 9318, which may suggest either a residual heterogeneity of the cell area taken, or perhaps that the tumour cells contain one normal and one mutant *TP53* copy. We consistently found in these experiments that the strength of the PCR signal from the 'normal allele' was greater than that from the mutant. By comparison, the prostate cancer cell line PC-3, showed a completely different band shift compared with the normal control (Figure 3). This cell line has been shown to contain only one allele of *TP53*, with a nonsense mutation at codon 138 (Isaacs *et al.*, 1991).

To determine if an observed band shift for *TP53* exons 5/6 is indicative of a mutation, the normal and abnormal bands were extracted from the SSCP gel and directly sequenced after a second amplification by PCR. In Figure 3 using the specimen 9318, the SSCP bands labelled 1 and 2 were checked for mutations. While band 1 did not show any abnormality within the sequence, a point mutation was revealed in band 2, considered as abnormal by comparison with the normal migration pattern of *TP53* exons 5/6 in SSCP. Other minor species, when reamplified and sequenced, were wild type, and presumably represent alternative minor conformations adopted in the SSCP gel. The mutation was detected at position 13 336 of the *TP53* gene, and results in a missense mutation glutamine→histidine at codon 192 within exon 6 of the TP53 protein (Figure 4).

The DNA processed to obtain the data reported above was prepared from freshly fixed and haematoylin-eosin-stained frozen sections. We have also successfully extracted well-conserved DNA, for the purpose of PCR/SSCP analysis, from sections which have been previously immunostained using standard avidin–biotin–peroxidase detection, counterstained with haematoxylin, mounted under commercially available aqueous mounting media and then demounted for final processing. These sections had been stored at room temperature for up to 6 months. Furthermore, formalin-fixed and paraffin-embedded sections also produced similar results to freshly frozen/fixed sections, although it is clear that freshly fixed tissues give superior results, particularly for the amplification of DNA fragments greater than 500 bp (not shown). DNA has also been recovered from 10-year-old archival tissue, but we have no doubt it is possible to extract DNA from much older stored tissues.

### Discussion

The purpose of this study was to develop a reliable molecular analysis using homogeneous cell populations selected from prostatic carcinomas, to avoid masking of tumour-specific genetic changes by normal and reactive cells within the tumour specimens. Despite careful biopsy technique and crude microdissections, it has been almost impossible to obtain homogeneous prostatic carcinoma tissue, to determine molecular events involved in tumour development and progression (Sarkar *et al.*, 1993).

Tissue heterogeneity makes it difficult to assess how many copies of an abnormal gene are present compared with the number of normal copies. This heterogeneity raises questions about the reliability of results obtained from whole tissue DNA preparations, in which the signal from mutant alleles may be masked by the presence of normal alleles, and the results of the reconstruction experiment (Figure 1) emphasise the fact that at least 20% of the DNA present in the preparation before PCR should be mutated in order to score positively in PCR/SSCP analysis. Since Bartsch *et al.* (1989) could find only an average of 23% of tumour cells within 50 dissected biopsies of prostate cancer, our results suggest that

**Figure 4** Direct sequencing of specific DNA strands after SSCP separation of mutant and normal alleles. (**a**) Agarose gel (1.5% GTG agarose) to separate the primary 407 bp PCR product generated from microdissected stromal and epithelial components of prostatic carcinoma. (**b**) Autoradiograph (16 h exposure) of SSCP in a 17% MDE gel to separate the normal and mutant alleles of *TP53*. Note that in the epithelial cells one of the mutant strands co-migrated with normal *TP53* band 1. Fragment 2 was therefore a putative mutant strand with altered mobility. (**c**) Confirmatory sequence analysis of purified band 1 from the (normal) stromal track on the SSCP gel and band 2 from the epithelial track. Note that direct sequencing of band 1 from the epithelial track did not give a mutant pattern on reamplification (data not shown), whereas a mutation was revealed in band 2 with an exon 6 point mutation at position 13 336 (G T).

most genetic alterations in prostate cancer might escape detection by standard molecular methods such as PCR/SSCP. Furthermore, when only one allele is carrying a mutation, as is often the case with p53, more than 40% of the cells present in the specimen used for DNA extraction must be from the tumour.

By developing a precise microdissection technique, we are therefore able to analyse archival biopsies from patients who initially present with benign prostatic disorders which may progress to malignancy. However, with increased precision, there is also the possibility of intra- and inter-sample cross contamination, which can be ignored in most larger scale analyses of human genes by PCR. Experience in the detection of latent viral infections (Maitland and Lynas, 1991), and minimal residual disease in leukaemia patients (Potter *et al.*, 1992) underlines this concern. We have taken every reasonable precaution to avoid contamination, including UV treatment by cryosectioning blades, individual sterile slides, sterile microneedles discarded after each microdissection, and individual PCR quality reagents used in dedicated class 2 facilities. We are therefore confident that the presence of a strong normal *TP53* allele signal in the SSCP analysis of dissected tumour tissues is not a result of cross-contamination. It could either be due to increased efficiency of amplification of the normal allele, or to the presence of normal cells, which are identical to the tumour cells by both histological and immunocytochemical analysis, within the microdissected mass. Despite its frequent involvement in human cancer, the existence of *TP53* mutation in prostate cancer remains unclear. Our experiments set out to study the feasibility of SSCP band shift as a method of measuring the frequency of mutation in the human *TP53* gene exons 5/6, by PCR/SSCP analysis of microdissected prostate tissues containing histopathologically documented tumour foci. We chose these exons for a preliminary study since they are frequently mutated in human cancer. According to Caron de Fromentel and Soussi (1992), exons 5 and 6 contain respectively, 28.5% and 12% of all p53 mutations, irrespective of the origin of the tumour.

Careful studies of documented tumour after microdissection should increase the observed frequency of many mutations, especially in prostate cancer when DNA abnormalities could neither be detected by crude DNA preparation and/or immunology. Our results emphasise the need for precise microdissection as part of a study of mutations in exons 5–8 of *TP53* where almost 97% mutations have been located in human cancer (Cariello *et al.*, 1994; Greenblatt *et al.*, 1994; Hollstein *et al.*, 1994). It should also remove the uncertain and inconsistent results obtained in many of the current mutation studies with this tumour and other histologically heterogeneous tumours (Noguchi *et al.*, 1992; Whetsell *et al.*, 1992). Such studies are of critical importance to establish the regulatory pathways which control stepwise progression leading to prostatic carcinoma.

**Acknowledgements**
The authors wish to thank Lucy Hopwood and Mike Anderson for technical support, Peter Crosby and Meg Stark for photographic facilities. We thank Drs J Brannigan and D Roper (Department of Chemistry, University of York) for synthesis of oligonucleotides. This work was supported by a programme grant from Yorkshire Cancer Research Campaign, UK. Dr Todor Dimitrov was an EC fellow, as part of the 'Go West' programme.

### References

BALL RK, SIEGL B, QUELHORST S, BRANDER G AND BRAUN DG. (1984). Monoclonal antibodies against simian virus 40 large T tumor antigen. Epitope mapping, papova virus cross reaction and cell surface. *EMBO J.*, **3**, 1485–1491.

BARTSCH G, MIKUZ G, DIETZE O AND ROHR HP. (1989). Morphometry in the abnormal growth of the prostate. In *The Prostate* Fitzpatrick JM and Krane RJ (eds) pp. 19–31. Churchill Livingstone: London.

BISHOP JM. (1991). Molecular themes in oncogenesis. *Cell*, **64**, 235-248.

BOOKSTEIN R, MACGROGAN D, HILSENBECK SG, SHARKEY F AND ALLRED DC. (1993). p53 Is mutated in a subset of advanced-stage prostate cancers. *Cancer Res.*, **53**, 3369-3373.

BUTTYAN R AND SLAWIN K. (1993). Rodent models for targeted oncogenesis of the prostate gland. *Cancer and Metastasis Reviews*, **12**, 11-19.

CARIELLO NF, BEROUD C AND SOUSSI T. (1994). Database and software for the analysis of mutations at the human p53 gene. *Nucleic Acids Res.*, **22**, 3549-3550.

CARON DE FROMENTEL C AND SOUSSI T. (1992). TP53 tumour suppressor gene: a model for investigating human mutagenesis. *Genes, Chrom. Cancer*, **4**, 1-15.

DINJENS WNM, VAN DER WEIDEN MM, SCHROEDER FH, BOSMAN FT AND TRAPMAN J. (1994). Frequency and characterization of p53 mutations in primary and metastatic human prostate cancer. *Int. J. Cancer*, **56**, 630-633.

EFFERT PJ, NEUBAUER A, WALTHER PJ AND LIU ET. (1992). Alterations of the p53 gene are associated with the progression of a human prostate carcinoma. *J. Urol.*, **147**, 789-793.

EFFERT PJ, MCCOY RH, WALTHER PJ AND LIU ET. (1993). p53 Gene alterations in human prostate carcinoma. *J. Urol.*, **150**, 257-261.

FEARON ER AND VOGELSTEIN B. (1990). A genetic model for colorectal tumorigenesis. *Cell*, **61**, 759-767.

GANNON JV, GREAVES R, IGGO R AND LANE DP. (1990). Activating mutations in p53 produce a common conformtational effect. A monoclonal antibody specific for the mutant form. *EMBO J.*, **9**, 1595-1602.

GLEASON DF. (1992). Histologic grading of prostate cancer: a perspective. *Hum. Pathol.*, **23**, 273-279.

GREENBLATT MS, BENNETT WP, HOLLSTEIN M AND HARRIS CC. (1994). Mutations in the *p53* tumor suppressor gene: Clues to cancer etiology and molecular pathogenesis. *Cancer Res.*, **54**, 4855-4878.

HARLOW E, CRAWFORD LV, PIM DC AND WILLIAMSON NM. (1981). Monoclonal antibodies specific for simian virus 40 tumor antigens. *J. Virol.*, **39**, 861-869.

HAYASHI K. (1991). PCR-SSCP: A simple and sensitive method for detection of mutations in genomic DNA. *PCR Methods Appl.*, **1**, 34-38.

HOLLSTEIN M, RICE K, GREENBLATT MS, SOUSSI T, FUCHS R, SORLIE T, HOVIG E, SMITH-SORENSEN B, MONTESANO R AND HARRIS CC. (1994). Database of p53 gene somatic mutations in human tumors and cell lines. *Nucleic Acids Res.*, **17**, 3551-3555.

ISAACS WB, CARTER BS AND EWING CM. (1991). Wild-type p53 suppresses growth of human prostate cancer cells containing mutant p53 alleles. *Cancer Res.*, **51**, 4716-4720.

LEVINE AJ, MOMAND J AND FINLAY CA. (1991). The p53 tumour suppressor gene. *Nature*, **351**, 453-456.

LISITSYN N, LISTSYN N. & WIGLER M. (1993). Cloning the differences between two complex genomes. *Science*, **259**, 946-951.

MAITLAND NJ AND LYNAS C. (1991). The detection of latent virus infection by polymerase chain reaction. In *Methods in Molecular Biology*, Vol. 9, *Protocols in Human Molecular Genetics*, Mathew C (ed.) pp. 347-364. Humana: Clifton, NJ. USA.

MEYERS FJ, CHI S-G, FISHMAN JR, DE VERE WHITE RW AND GUMERLOCK PH. (1993). p53 mutations in benign prostatic hyperplasia. *J. Natl Cancer Inst.*, **85**, 1856-1858.

MILNER J, COOK A AND SHELDON M. (1987). A new p53 antibody, previously reported to be directed against the large T antigen of simian virus 40. *Oncogene*, **1**, 453-455.

NAVONE NM, TRONCOSO P, PISTERS LL, GOODROW TL, PALMER JL, NICHOLS WW, VON ESCHENBACH AC AND CONTI CJ. (1993). p53 Protein accumulation and gene mutation in the progression of human prostate carcinoma. *J. Natl Cancer Inst.*, **85**, 1657-1669.

NOGUCHI S, MOTOMURA K, INAJI H, IMAOKA S AND KOYAMA H. (1992). Clonal analysis of human breast cancer by means of the polymerase chain reaction. *Cancer Res.*, **52**, 6594-6597.

POTTER MN, STEWARD CG, MAITLAND NJ AND OAKHILL A. (1992). Detection of clonality in childhood B-lineage acute lymphoblastic leukaemia by the polymerase chain reaction. *Leukemia*, **6**, 289-294.

SARKAR FH, SAKR WA, LI Y-W, SREEPATHI P AND CRISSMAN JD. (1993). Detection of human papillomavirus (HPV) DNA in human prostatic tissues by polymerase chain reaction (PCR). *Prostate*, **22**, 171-180.

SINGER-SAM J, TANGUAY RL AND RIGGS AD. (1989). Use of Chelex to improve the PCR signal from a small number of cells. *Amplifications*, **3**, 11.

SOINI Y, PAAKKO P, NUORVA K, KAMEL D, LANE DP AND VAHAKANGAS K. (1992). Comparative analysis of p53 protein immunoreactivity in prostatic, lung and breast carcinomas. *Virchows Arch. A Pathol. Anat. Hispathol.*, **421**, 223-228.

UCHIDA T, WADA C, SHITARA T, EGAWA S AND KOSHIBA K. (1993). Infrequent involvement of *p53* gene mutations in the tumorigenesis of Japanese prostate cancer. *Br. J. Cancer*, **68**, 751-755.

VAN VELDHUIZEN PJ, SADASIVAN R, GARCIA F, AUSTENFELD MS AND STEPHENS RL. (1993). Mutant p53 expression in prostate carcinoma. *Prostate*, **22**, 23-30.

VISĀKORPI T, KALL HEIKKINEN A, KOIVULA T AND ISOLA J. (1992). Small subgroup of aggressive, highly proliferative prostatic carcinomas defined by p53 accumulation. *J. Natl Cancer Inst.*, **84**, 883-887.

VOELLER HJ, SUGARS LY, PRETLOW T AND GELMANN EP. (1994). p53 oncogene mutations in human prostate cancer specimens. *J. Urol.*, **151**, 492-495.

VOJTESEK B, BARTEK J, MIDGLEY CA AND LANE DP. (1992). An immunochemical analysis of the human nuclear phosphoprotein p53. New antibodies and epitope mapping using recombinant p53. *J. Immunol. Meth.*, **151**, 237-244.

WHETSELL L, MAW G, NADON N, RINGER DP AND SHAEFER FV. (1992). Polymerase chain reaction microanalysis of tumours from stained histological slides. *Oncogene*, **7**, 2355-2361.

ZHANG L, CUI X, SCHMITT K, HUBERT R, NAVIDI W AND ARNHEIM N. (1992). Whole genome amplification from a single cell: implications for genetic analysis. *Proc. Natl Acad. Sci. USA*, **89**, 5847-5851.